# Optimization of a Screw Centrifugal Blood Pump Based on Random Forest and Multi-Objective Gray Wolf Optimization Algorithm

**DOI:** 10.3390/mi14020406

**Published:** 2023-02-08

**Authors:** Teng Jing, Haoran Sun, Jianan Cheng, Ling Zhou

**Affiliations:** Research Center of Fluid Machinery Engineering & Technology, Jiangsu University, Zhenjiang 212013, China

**Keywords:** centrifugal blood pump, random forest (RF), numerical simulation, multi-objective gray wolf optimization (MOGWO), hemolysis, scalar shear stress

## Abstract

The centrifugal blood pump is a commonly used ventricular assist device. It can replace part of the heart function, pumping blood throughout the body in order to maintain normal function. However, the high shear stress caused by the impeller rotating at high speeds can lead to hemolysis and, as a consequence, to stroke and other syndromes. Therefore, reducing the hemolysis level while ensuring adequate pressure generation is key to the optimization of centrifugal blood pumps. In this study, a screw centrifugal blood pump was used as the research object. In addition, pressure generation and the hemolysis level were optimized simultaneously using a coupled algorithm composed of random forest (RF) and multi-objective gray wolf optimization (MOGWO). After verifying the prediction accuracy of the algorithm, three optimized models were selected and compared with the baseline model in terms of pressure cloud, 2D streamline, SSS distribution, HI distribution, and vortex distribution. Finally, via a comprehensive evaluation, the optimized model was selected as the final optimization design, in which the pressure generation increased by 24% and the hemolysis value decreased by 48%.

## 1. Introduction

Heart failure (HF) is a cardiovascular disease with high morbidity and mortality [1]. According to relevant studies [2,3], there are more than 64.3 million heart failure patients worldwide, and the incidence of heart failure is increasing with global population growth. Thus, it represents a serious issue for the global healthcare economy. For end-stage heart failure, heart transplantation is an effective treatment. However, the severe shortage of donors is an issue, and the long waiting times mean that a large number of patients miss the optimal time for heart transplantation. In addition, the increasing gap between the number of heart donors and heart failure patients is gradually increasing the mortality rate [4,5]. For patients with end-stage heart failure who are unable to undergo heart transplantation and are not suitable, a ventricular assist device (VAD) becomes the last option to significantly improve their chances of survival and their quality of life [6,7,8,9]. Centrifugal blood pumps are a common life support device [10] and are widely used in left heart assist devices and extracorporeal membrane pulmonary oxygenation (ECMO) [11,12]. These operations are characterized by the need for a high rotational speed in order to assist the left ventricle to return to a normal pumping volume [13]. Under high rotational speed, high shear stresses are generated near the impeller and at various gaps in the pump body [14]. This causes a large number of erythrocytes to rupture, which initiates the release of hemoglobin from the cells into the plasma, provoking hemolysis [15]. This also induces acquired vascular hemophilia and platelet activation [16,17], which can be life-threatening in severe cases. In addition, prolonged contact between the blood and foreign surfaces can cause platelets to activate and aggregate, forming clots [18]. Current methods of reducing blood damage are usually achieved by optimizing the impeller structure to improve efficiency or by lowering the speed to reduce shear stress. Reducing the level of hemolysis while ensuring a sufficient head value is the main focus of centrifugal blood pump design and optimization.

During the development of blood pumps, designers usually ensure that the impeller has an irregular geometry according to the hemodynamics of the blood flow. The complex modeling process has led researchers to vary one or several parameters by manually selecting values during impeller optimization and comparing hydraulic performance with HI indices [19,20,21,22]. However, relying on empirical changes in parameters without validation may cause significant degradation of the centrifugal blood pump performance and can even damage the equipment. Therefore, various scholars have applied machine-learning-based optimization methods to such engineering problems and achieved reductions in the HI level. For example, Onder et al. changed the blade tip clearance and the axial clearance between the impeller and the pump, establishing the optimal clearance width using the artificial bee colony (BA) algorithm, which resulted in a 42% reduction in the wall shear stress [23]. Antaki et al. reduced the formation of thrombus and the occurrence of HI conditions by optimizing the shape of the blood path [24]. In fact, a change in one of the structural parameters of the impeller can simultaneously have a positive or negative impact on blood pump efficiency and the HI level. For such multi-objective engineering problems, multi-objective optimization algorithms are one of the reliable and effective methods [25], as decision makers can select the optimal solution from a wide range of solutions according to their actual needs. For example, Behnam Ghadimi et al. performed complete multi-objective optimization on a centrifugal left heart assist device, HeartMateIII, using a multi-objective genetic algorithm [17]. They established 11 variable points to simultaneously control the blade and volute profile of HeartMateIII. With the help of artificial neural networks (ANNs), they established the mapping relationship between each variable point and the optimization objective, which was used as a proxy model in combination with a fast and elitist non-dominated multi-objective genetic algorithm (NSGA-II) to achieve optimization. The final efficiency was improved by 6.1% while the HI level decreased by 14.7%. In addition, Zhu et al. [26] used 14 design variables to control the axial blood pump diffuser blade shape combined with a genetic algorithm to find the Pareto optimal solution set to achieve multi-objective optimization in order to increase the pressure output and reduce the reflux index. The majority of optimization algorithms currently used by researchers are more traditional and are not applied to spiral centrifugal pumps, which have superior biocompatibility. In terms of optimization algorithms, this study used the multi-objective gray wolf optimization (MOGWO) algorithm, which is more suitable for engineering optimization problems, as an optimization tool for a screw centrifugal blood pump. In addition, the random forest prediction algorithm, which is more suitable for smaller sample sizes, was used to predict the pressure output and hemolysis value of the blood pump. With this, we were able to improve prediction accuracy and reduce the time costs associated with data collection.

In this study, a self-developed screw centrifugal blood pump was used as the research object, and several optimization methods were established, including computational fluid dynamics (CFD), the Latin hypercube sampling method, the random forest prediction algorithm (RF), and the multi-objective gray wolf optimizer (MOGWO), and the pressure generation and HI performance of the screw centrifugal blood pump were simultaneously optimized. Three models were selected among many optimal solutions for the comparative analysis of pressure generation and HI levels.

## 2. Materials and Methods

### 2.1. Optimization Process

**Figure 1:**
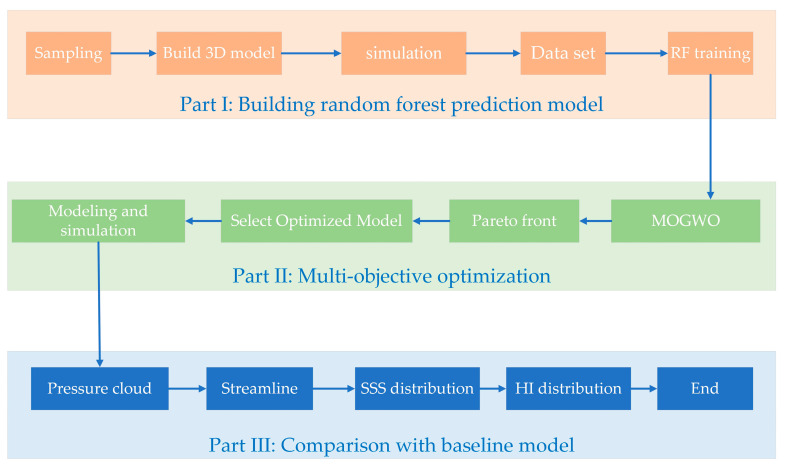
Optimization process.

The overall optimization process for the blood pump is divided into three parts (Figure 1). The first step is the creation of a random forest prediction model. First, the parameters and boundary values controlling the impeller shape are determined. Then, 240 sets of impeller parameters are randomly obtained using the Latin hypercube sampling method, and a 3D model is built and numerically simulated to obtain a database consisting of impeller parameters and pressure generation, and HI values. Thereafter, the random forest prediction model is trained. The second part is multi-objective optimization, using the multi-objective gray wolf optimizer (MOGWO) to calculate the Pareto front. This involves selecting three different optimized models in the Pareto front for modeling and performing the numerical simulation. In the third part, the internal flow differences between the baseline model and the optimized model are analyzed, and the model with the best-combined situation is selected. Each step is described carefully in the following section.

#### 2.1.1. Model Introduction and Parameterization

**Figure 2:**
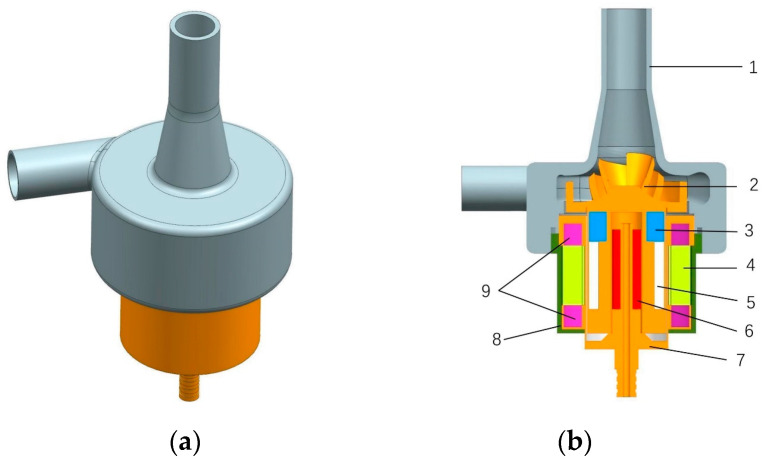
Three-dimensional view and cross-sectional view of the screw centrifugal pump. (**a**) Three-dimensional view of the screw centrifugal pump. (**b**) Cross-sectional view and internal structure of the screw centrifugal pump. 1—house; 2—impeller; 3—magnet location sleeve; 4—coil winding; 5—magnet; 6—bearing; 7—motor inner casing; 8—motor housing; 9—locating sleeve.

For this study, we used a self-developed screw centrifugal pump as the optimized object, because a screw centrifugal pump combines the advantages of volumetric pumps and centrifugal pumps [27]. The impeller was designed with a unique three-dimensional screw structure, which has superior nondestructive performance, a wider high-efficiency range, and better non-clogging characteristics than ordinary centrifugal pump impellers [28,29]. These advantages of the screw centrifugal pump reduce the amount of damage to the blood. A three-dimensional diagram and a cross section of the baseline model pump are shown in Figure 2.

**Figure 3:**
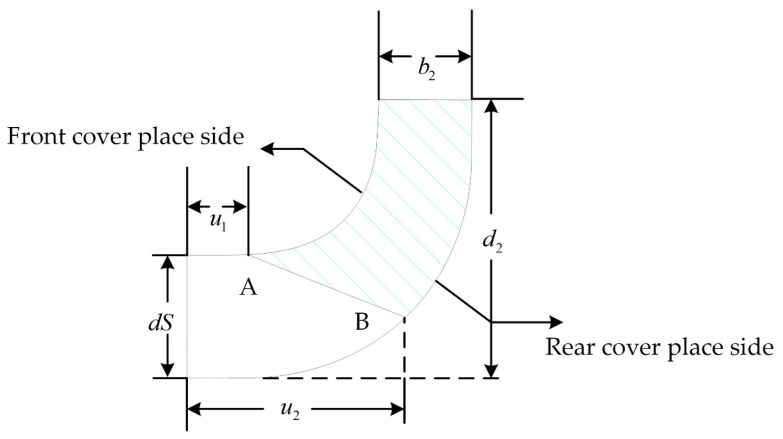
Median diagram of the impeller.

In the design process of centrifugal pumps, the axial projection diagram of the impeller is crucial. This is because a large number of parameters in the diagram can control the shape of the impeller [30,31] and changing any of these parameters will deform the impeller. This provides very convenient conditions for impeller parameterization. Figure 3 is the axial projection of the baseline model impeller. The shaded area in the figure represents the shape of the blade axis surface. Moreover, $d_{2}$ is the impeller’s outer diameter. In this study, this parameter was fixed at 30 mm. In addition, dS denotes the impeller inlet diameter, $b_{2}$ denotes the blade exit width, and the two circular curves represent the front cover line of the impeller and the rear cover line of the impeller, respectively. The straight line running from A to B is the position of the blade inlet side and it is defined by $u_{1}$ and $u_{2}$, which denote the radial length from the intersection of the blade inlet edge line with the front and rear cover profiles to the impeller inlet, respectively. It can be seen that the position of the blade inlet edge is controlled by $u_{1}$ and $u_{2}$ together. There are certain impeller parameters that cannot be described visually in the axial projection diagram, such as the blade wrap angle (ϕ), the blade exit angle ($\beta_{2}$), and the axis ratio of the leading edge of the blade ($A_{r}$). These are also part of impeller parameterization as they have an impact on the impeller shape, hydraulic performance, and hemolysis performance of the blood pump [32,33].

**Figure 4:**
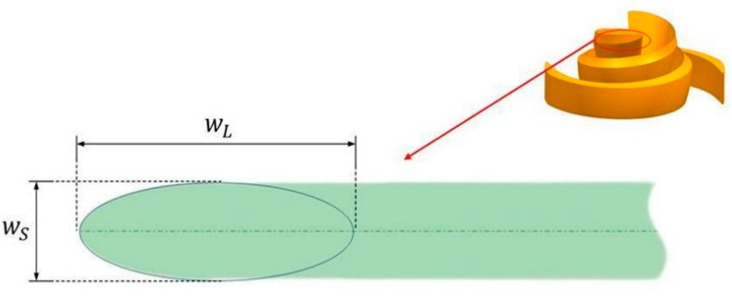
Axis ratio of blade leading edge.

It is worth mentioning that, in this study, we used an elliptical leading-edge design scheme [34]. The top view of the blade’s leading-edge shape is shown in Figure 4. The sharpness of the ellipse is expressed by Equation (1):

(1) $$A_{r}=\frac{w_{L}}{w_{S}}$$

where $w_{L}$ and $w_{S}$ represent the long and short axes of the ellipse, respectively. The blade thickness increases linearly from 1 mm at the leading edge ($w_{S}$) to 2 mm at the trailing edge, and the axial thickness remains constant. The inlet and outlet diameters of the volute are 10 mm. Table 1 summarizes all the parameter values of the baseline model and their variation ranges, where $x_{L}$ and $x_{U}$ represent the lower and upper bounds of the impeller parameters, respectively.

#### 2.1.2. Establishing the Database

When building proxy models for expensive functions or experimental data, the pre-experimental design is particularly important for the selection of the initial sampling points. Thus, how to achieve good space-filling effects with as few samples as possible is a hot research topic. In computer experiments, classical sampling methods include Monte Carlo (MCS), Latin hypercube sampling (LHS), Sobol sequence, etc. [35]. Among them, Latin hypercube sampling is popular. It is a random sampling method that was improved by Mckay et al. [36,37] based on the Monte Carlo sampling method. They enhanced a core sampling strategy with stratified sampling, which ensures full coverage of the multivariate distribution and accuracy, while significantly reducing the sample size. In this study, 240 sets of impeller parameters were randomly generated using the Latin hypercube sampling method in the range of impeller parameter variation. Thereafter, all of them were modeled and numerically simulated to obtain a database consisting of impeller parameters and head and HI values.

#### 2.1.3. Numerical Simulation and Boundary Conditions

**Figure 5:**
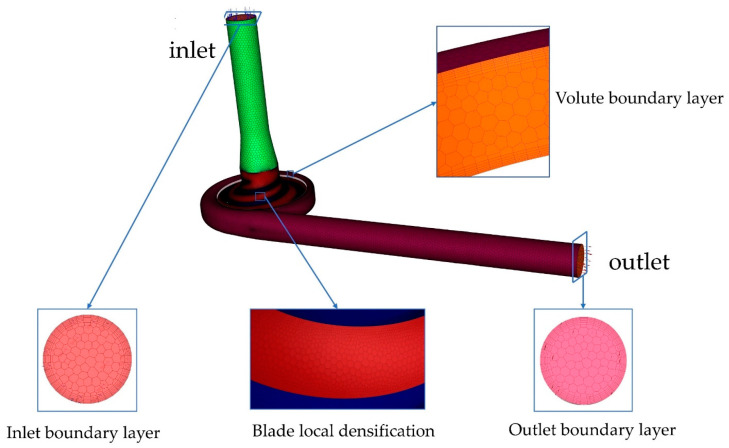
Computational domain meshes.

The model for the area calculation used for the numerical simulation is shown in Figure 5. The whole stream field area was divided into three areas: the inlet domain, the impeller domain, and the volute domain. The structure of the inlet domain and volute domain is relatively simple and can be drawn manually using the 3D modeling software NX12.0. The impeller domain was automatically generated using CFTurbo2021 R2.2 by inputting parameters.

In this study, Fluent Meshing, a fast and high-quality pre-processing meshing software, was used to discretize the computational domain. High-adaptability polyhedral mesh was used inside the computational domain. Six-layer structured mesh was used for the walls. In addition, the mesh of the blade wall was encrypted to ensure a more accurate calculation of flow details. Finally, the orthogonal qualities of the meshes were all greater than 0.3, and $y^{+}$ near the wall was less than 5, conforming to SST K−ω turbulence modeling standards. To reduce computational costs, the mesh independence of the baseline model was verified at a speed of 3900 rpm and a flow rate of 5 L/min. The mesh-independence verification carried out is presented in this paper. Table 2 shows that when the number of meshes was more than 3,825,107, the pressure generation value no longer changed. To reduce the simulation time, the number of meshes in option 3 was chosen.

The finite element analysis software Fluent (Ansys, Canonsburg, PA, USA) was used as the solver for the numerical simulations in this paper. Under high shear stress, blood can usually be considered an incompressible Newtonian fluid with a viscosity of 0.0035 kg/m.s and a density of 1050 kg/$m^{3}$ [38,39,40]. Flow in the pump is controlled by the Reynolds averaged Navistokes (RANS) equations [41]. In this study, steady-state simulations were performed using the SST K−ω turbulence model with dual controlling equations [42,43]. The control equation of the SST K−ω turbulence model is shown in Equation (2):

(2) $$\left\{ \begin{array}{l} \rho \frac{\partial k}{\partial t}+\rho \bar{u_{j}}\frac{\partial k}{\partial x_{j}}=P_{k}-\rho \beta_{*}\omega k+\frac{\partial }{\partial x_{j}}\left[\left(\mu +\frac{\mu_{t}}{\sigma_{k}}\right)\frac{\partial k}{\partial x_{j}}\right]\\ \rho \frac{\partial \omega }{\partial t}+\rho \bar{u_{j}}\frac{\partial \omega }{\partial x_{j}}=\alpha P_{\omega }-\rho \beta \omega^{2}+\frac{\partial }{\partial x_{j}}\left[\left(\mu +\frac{\mu_{t}}{\sigma_{\omega }}\right)\frac{\partial \omega }{\partial x_{j}}\right]+2\rho \left(1-F_{B}\right)\frac{1}{\sigma_{\omega_{out}}}\frac{\partial k}{\partial x_{j}}\frac{\partial \omega }{\partial x_{j}} \end{array}\right.$$

where ω is the unknown specific dissipation rate; $\mu_{t}$ is the eddy viscosity; $F_{B}$ is the mixing function; $\beta_{*}=0.09$; and $\sigma_{\omega_{out}}=1.168$.

According to the flow rate of 5 L/min and the inlet cross-sectional area, the blood pump inlet was set to a velocity inlet boundary condition of 1.061 m/s, the blood pump outlet was set to the outflow outlet condition, the rotational speed was controlled at 3900 rpm, the impeller walls were set as rotating walls, and the remaining walls were assumed to be slip-free. The computational domain contains one rotational domain and two stationary domains. Based on the numerical simulation study of the blood pump [17,44,45,46,47], the multiple reference coordinate system (MRF) was selected, in which the impeller domain was the rotational coordinate system, and the inlet domain and the worm-casing domain were the stationary coordinate systems. The surfaces overlapping between the three computational domains were set as interfaces. The semi-implicit SIMPLEC algorithm was used to solve the pressure–velocity-coupled equations, and the convergence criterion was set to $10^{-5}$. After spatial discretization, all equations were set as second-order windward equations.

#### 2.1.4. Calculation of Pressure Generation, Scalar Shear Stress, and HI Value

The calculation of the pressure generation in this study is as follows:

(3) $$H=\frac{p_{out}-p_{in}}{\rho g}$$

where *H* represents the pressure generation, $p_{out}$ denotes the outlet pressure (Pa), $p_{in}$ denotes the inlet pressure (Pa), and ρ is the blood density.

The Lagrangian method and the power-law model based on scalar shear force and red blood cell exposure time proposed by Giersiepen et al. [48] were used to calculate the intra-pump hemolysis values. The mathematical formulation of the model is as follows:

(4) $$HI=\frac{dHb}{Hb}=Ct^{\alpha }\tau^{\beta }$$

where Hb is total hemoglobin, dHb is total free hemoglobin, C, α, β are the empirical constants ($C=3.62\times 10^{-7},\;\alpha =0.785,\;\beta =2.418$), t is the erythrocyte exposure time, and τ is the scalar shear stress (later referred to as SSS). SSS is the sum of the viscous shear stress ($\sigma_{ij}$) and Reynolds shear stress ($\rho \bar{u_{i}}\bar{u_{j}}$), i.e.,

(5) $$\tau_{ij}=\sigma_{ij}+\rho {\bar{u}}_{i}{\bar{u}}_{j}$$

In conjunction with the SSS calculation proposed by Bludszuweit [49],

(6) $$\tau ={\left(\frac{1}{6}{\sum \left(\tau_{ii}-\tau_{jj}\right)}^{2}+\sum \tau_{ij}^{2}\right)}^{\frac{1}{2}}$$

The final expression of SSS takes the following form:

(7) $$\tau =\frac{1}{3}(\tau_{11}^{2}+\tau_{22}^{2}+\tau_{33}^{2}-\tau_{11}\tau_{22}-\tau_{11}\tau_{33}-\tau_{22}\tau_{33})+\tau_{12}^{2}+\tau_{23}^{2}+\tau_{13}^{2}$$

#### 2.1.5. Random Forest (RF)

**Figure 6:**
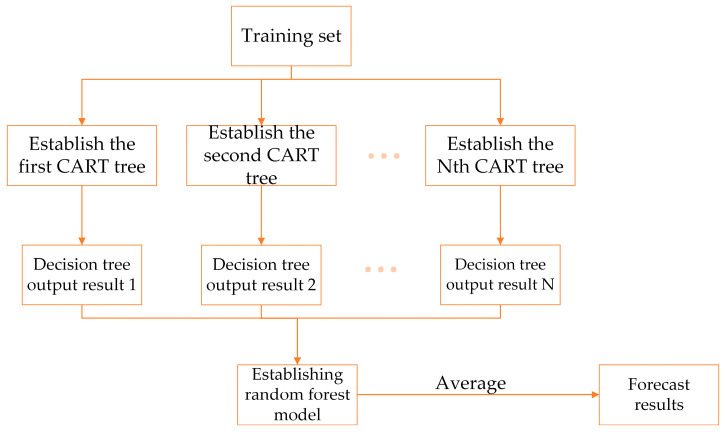
Prediction principle of random forest.

Random forest (RF) was proposed by Leo Breiman [50] in 2001. As shown in Figure 6, it is a prediction algorithm that combines bagging integrated learning theory with a random subspace. Its core lies in N CART (classification and regression trees) composed of $g(D,\theta_{n})$. The final prediction result can be obtained by averaging the regression results of all regression trees, which means the random forest algorithm is more resistant to overfitting, has a lower error rate [51], and is more accurate when making predictions on small samples.

In this study, we randomly selected 200 sets of data from the database mentioned above and used them as the training set. In addition, we trained and built two independent random forest proxy models to predict pressure generation and HI. The input values of the proxy models were the impeller parameters, the output values were HI and pressure generation, and the rest of the data were used as the prediction set to evaluate the prediction accuracy of the models.

To quantitatively evaluate the prediction accuracy of the random forest prediction models, in this study, mean absolute percentage error (MAPE) [52] and mean square error (MSE) [53] were used as model accuracy evaluation metrics for the HI prediction set and the pressure generation prediction set. MAPE and MSE are calculated as follows:

(8) $$\mathrm{MAPE}=\frac{1}{n}\sum_{i=1}^{n}\left|\frac{{\hat{y}}_{i}-y_{i}}{y_{i}}\right|$$

(9) $$\mathrm{MSE}=\frac{1}{n}\sum_{i=1}^{n}{\left({\hat{y}}_{i}-y_{i}\right)}^{2}$$

where $y_{i}$ and ${\hat{y}}_{i}$ are the true and predicted values of the *i*-th sample, $\bar{y}$ is the average of the actual values of the samples, and n is the capacity of the corresponding sample.

**Figure 7:**
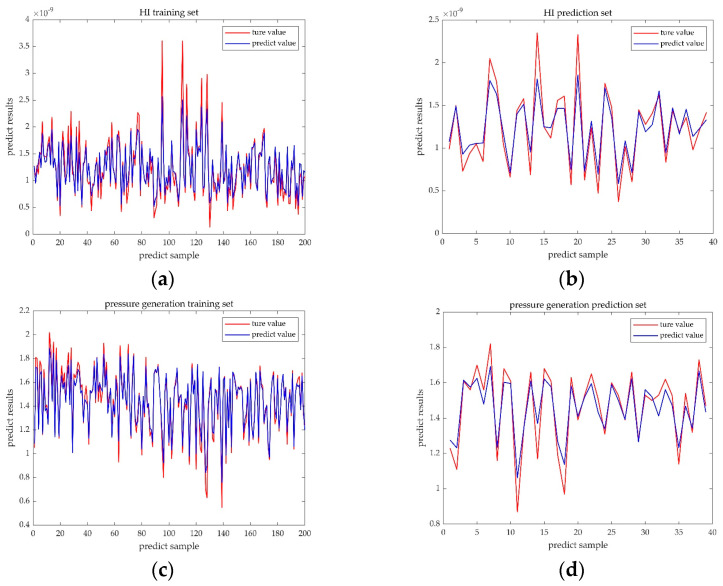
Comparison of the RF predictive value and true value. (**a**) Comparison result of the HI training set. (**b**) Comparison result of the HI prediction set. (**c**) Comparison result of the pressure generation training set. (**d**) Comparison result of the pressure generation prediction set.

Figure 7 shows the predicted and true values of the random forest algorithm, where the blue curve represents the predicted value and the red curve represents the true value. The curve trends of the prediction set and the training set are consistent with a high degree of similarity, with some errors existing at extreme values only. The quantitative model accuracy evaluation is shown in Table 3.

In Table 3, the MSE and MAPE of both the pressure generation training set and the HI training set were maintained below 0.5, which demonstrated that the random forest model has a high prediction accuracy and can accurately regress the mapping relationship between the design parameters and the pressure generation and HI levels.

#### 2.1.6. Multi-Objective Gray Wolf Optimization Algorithm (MOGWO)

The gray wolf optimization algorithm (GWO) is a pack intelligence optimization algorithm designed by Mirjalili [54]. It was inspired by the social stratification characteristics and hunting and trapping behavior of wolves. It has the advantages of strong convergence performance, a simple structure, and easy implementation. There exist convergence factors with self-adaptive adjustment and information feedback mechanisms. Thus, a balance between local optimization and global optimization is achieved. For this reason, it has good problem-solving accuracy. Gray wolves are divided into four social classes: α, β, δ, ω. After sorting according to the adaptation function, α wolves represent the optimal solutions. β wolves and δ wolves represent the suboptimal solutions, with their role to assist α wolves in making decisions. The remaining candidate solutions are defined as ω wolves. Classes of α wolves, β wolves, and δ wolves command the hunting behavior, and ω wolves follow the above-mentioned higher-level wolves in hunting. Since the position of the prey (the optimal solution) in the solution space is not known, it is assumed that the positions of wolf α, wolf, and wolf δ are closest to the prey.

**Figure 8:**
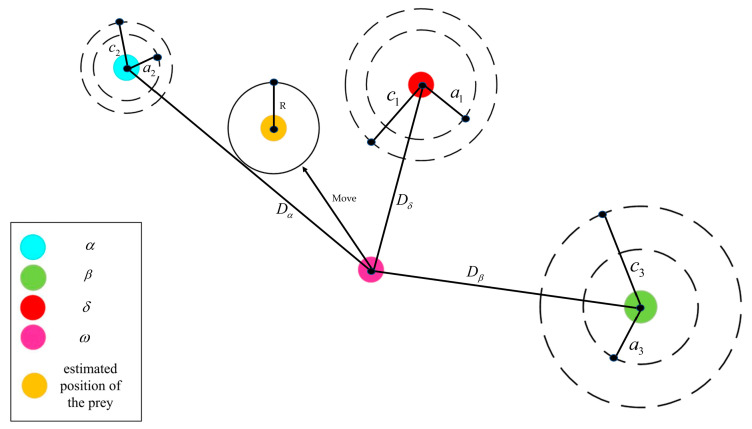
Schematic diagram of gray wolf location update [54].

As shown in Figure 8 [54], the hunting process of the gray wolf surrounding prey is represented by the following mathematical model:

(10) $$\vec{D}=\left|\vec{C}\cdot {\vec{X}}_{P}(t)-\vec{X}(t)\right|$$

(11) $$\vec{X}(t+1)={\vec{X}}_{p}(t)-\vec{A}\cdot \vec{D}$$

where $\vec{C}$ and $\vec{A}$ are coefficient vectors determined by random values, $\vec{X_{p}}\left(t\right)$ and $\vec{X}\left(t\right)$ represent the position vector of the prey and the position vector of the gray wolf at iteration up to the t-th generation, respectively [54]. Since the location of the optimal solution in the solution space is not known, it is assumed that the locations of wolf α, wolf β, and wolf δ are closest to the optimal solution. After recording the positions of these three wolves, the ω wolf is ordered to approach the α wolf, and the β wolf is ordered to approach the δ wolf. During each iteration, the positions of the α wolf, the β wolf, and the δ wolf are updated via the formulae shown in Equation (12) [54]:

(12) $${\vec{D}}_{\alpha }=\left|{\vec{C}}_{1}\cdot {\vec{X}}_{\alpha }-\vec{X}\right|\;{\vec{D}}_{\beta }=\left|{\vec{C}}_{2}\cdot {\vec{X}}_{\beta }-\vec{X}\right|\;D_{\delta }=\left|{\vec{C}}_{3}\cdot {\vec{X}}_{\delta }-\vec{X}\right|$$

(13) $${\vec{X}}_{1}={\vec{X}}_{\alpha }-{\vec{A}}_{1}\cdot ({\vec{D}}_{\alpha })\;{\vec{X}}_{2}={\vec{X}}_{\beta }-{\vec{A}}_{2}\cdot ({\vec{D}}_{\beta })\;{\vec{X}}_{3}={\vec{X}}_{\delta }-{\vec{A}}_{3}\cdot ({\vec{D}}_{\delta })$$

Averaging the positions of the α wolf, the β wolf, the δ wolf, and the result obtained is regarded as the final position after this iteration is updated. When $\left|\vec{A}\right|<1$ in Equation (12), the next generation of gray wolves can be located anywhere near the prey. The constant repetition of this behavior is to hunt the prey. It is worth mentioning that in order to prevent falling into local optimal solutions during the optimization process, the gray wolf chooses to move away from the prey when $\left|\vec{A}\right|>1$.

To enable the gray wolf optimization algorithm to perform multi-objective optimization, Mirjalili provided two components [55]. One is a storage component responsible for storing non-dominated Pareto optimal solutions, which implements the storage function of several optimal solutions. The other is a leader-selection strategy component, which can obtain dominance relations with the help of the Pareto global optimum concept but cannot compare solutions that are not dominated by each other. This component selects a new leader in the uncrowded region of non-dominated solutions according to the roulette wheel method, marked as α, β, δ, which is then archived.

In this study, two objective functions were chosen as constraints and the expressions are as shown in Equation (14):

(14) $$\begin{array}{l} Minimize:f_{1}(x)=-P\\ Minimize:f_{2}(x)=HI\;x={\left[x_{1},x_{2},\ldots ,x_{6}\right]}^{T},x\in (x_{L},x_{U}) \end{array}$$

where *P* and *HI* represent the random forest HI prediction model and the random forest indentation pressure generation prediction model, respectively, $x_{i}\left(i=1,2,\ldots ,6\right)$ represents the *i*-th design parameter, and $x_{L}$ and $x_{U}$ are the upper and lower bounds of each design parameter, respectively. All codes in this paper were written in the MATLAB environment, the number of populations in each iteration was set to 100, and the maximum number of external archives was chosen to be 100, for a total of 400 iterations, running on a PC with Inlet(R) Core(TM) i7-12700 k CPU 5.00 GHz 32 GB RAM.

## 3. Results

**Figure 9:**
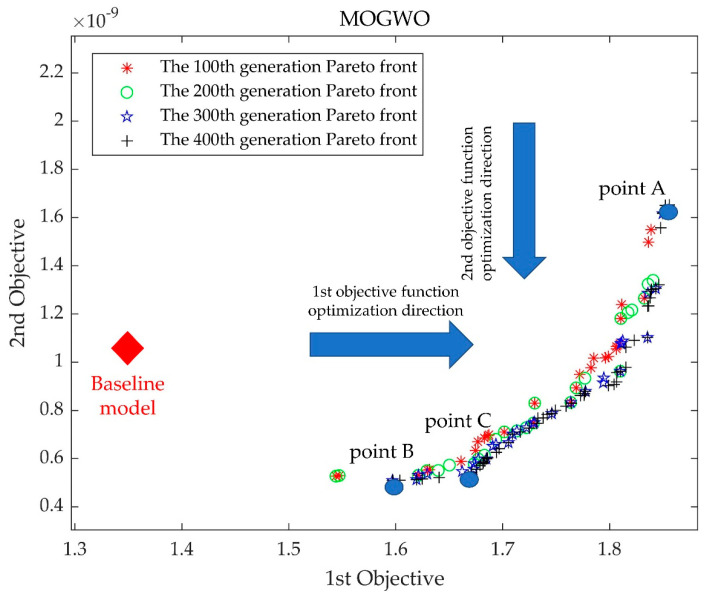
Paradoxical relationships between objective functions and the first 400 generations of the Pareto front.

**Figure 10:**
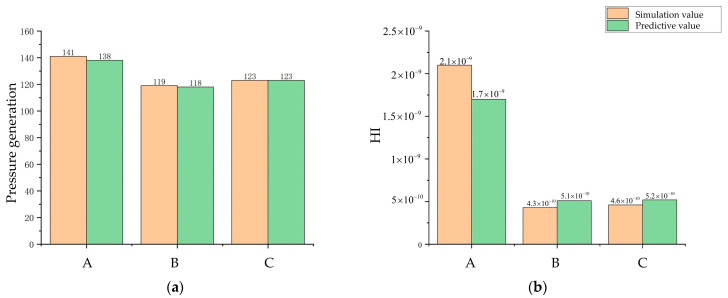
Algorithm prediction accuracy evaluation. (**a**) Comparison of the pressure generation prediction and the pressure generation simulation. (**b**) Comparison of the HI predictive value and the HI simulation value.

Figure 9 depicts the baseline model position and the process of Pareto front optimization at every 100th generation for the first 400 generations. It can be seen that the maximization of pressure generation and the minimization of the HI index were contradictory, and there were uneven distributions of non-dominated solutions in the Pareto frontier of different degrees in the first 300 generations, which creates the possibility of missing the optimal solution. This situation improved when it was iterated to 400 generations. Therefore, three optimized model pump models in the 400th generation of the Pareto front were selected for comparison with the baseline model pump, where point A is the first objective function optimal solution point (the point of the highest-pressure generation) and point B is the second objective function optimal solution point (the point of the lowest HI value). Point C is the intermediate point near point B. To verify the prediction accuracy of the random forest and MOGWO models, a numerical simulation was performed for models A, B, and C. The simulated and predicted values of each objective function for points A, B, and C are shown in Figure 10. The results show that the predicted values of the three optimized models are in good agreement with the simulation values, so the prediction using the random forest algorithm can be considered a reliable method.

### 3.1. Impeller Parameter Analysis

The impeller parameters of the four models before and after optimization are shown in Table 4. The pressure generation at point A increased by 41% as compared with the baseline model, but the HI increased by 105%. The axis ratio ($A_{r}$) did not change significantly as compared with the benchmark model, but the wrap angle decreased by 27 degrees and the blade outlet width increased by 1.1 mm. In addition, the A-point model exhibited the largest blade outlet width and exit blade angle. Therefore, the area of blood worked by the blades was larger than that of all the other model pumps. In terms of the blade inlet edge position, the A-point model $u_{2}$ value was greatly reduced, which means the blade inlet edge along the rear cover plate profile produced a large forward extension, the blade shape was distorted, and the hub side shrank significantly toward the center.

B-point model pressure generation was improved by 19% as compared with the base model, and HI was reduced by 50%. At this time, the B-point model wrap angle was reduced by 60° and the $u_{1}$ and $u_{2}$ values were lower than the base model. This allowed for the blade inlet side to be moved forward and positioned more axially. The blade exit width of the B-point model increased by 0.3 mm, which located the area where the blade worked on the blood and pressure generation between the baseline model pump and the A-point model pump. In terms of the wrap angle, the B-point model exhibited the most significant change, decreasing by 60° as compared with the baseline model, while the shaft ratio and blade exit did not exhibit a significant change.

The pressure generation of the C-point model was improved by 24%, and the HI value was reduced by 48%. The parameters of the C-point model and the B-point model were similar, with the main differences being related to the blade outlet width. Specifically, the blade exit width of the C-point model was slightly larger than that of the B-point model. The larger work area and surface area resulted in a simultaneous increase in pressure generation and the HI value; however, as can be seen from Figure 10, the increase in the HI value for the C-point model was almost negligible, while there was a significant increase in the pressure generation value.

### 3.2. Pressure Analysis

**Figure 11:**
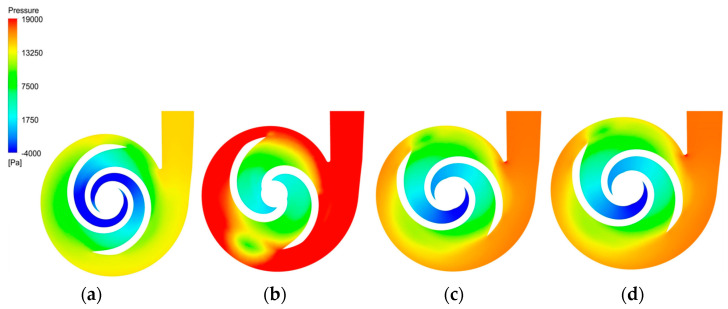
Pressure distribution clouds. (**a**) Pressure cloud of the baseline model. (**b**) Pressure cloud of the A−point model. (**c**) Pressure cloud of the B−point model. (**d**) Pressure cloud of the C−point model.

Figure 11 depicts the pressure distribution clouds of the baseline model and the optimized model in the XY direction. All models had a low-pressure zone at the impeller inlet, with the impeller diameter gradually increasing the pressure and finally reaching its maximum at the trailing edge of the blade and the volute. The optimized pressure values at the outlet of all models were improved to different degrees, with the largest pressure of 25,028 Pa being observed at the outlet of the A-point model, although there was a region of uneven pressure distribution at the volute. The pressures at the outlet of the B-point model and C-point model were 19,535 Pa and 19,679 Pa, respectively. Both exhibited steady pressure growth, with no obvious excessive pressure gradient area, but the outlet pressure value of the C-point model was slightly higher than that of the B-point model. Therefore, the C-point model can provide higher pressure generation than the B-point model under the same operating conditions.

### 3.3. Stream Field Analysis

**Figure 12:**
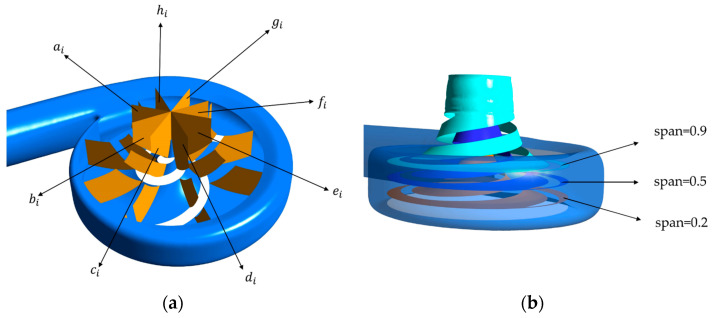
Streamline monitoring surfaces. (**a**) Axial monitoring surface. (**b**) Radial monitoring surface.

**Figure 13:**
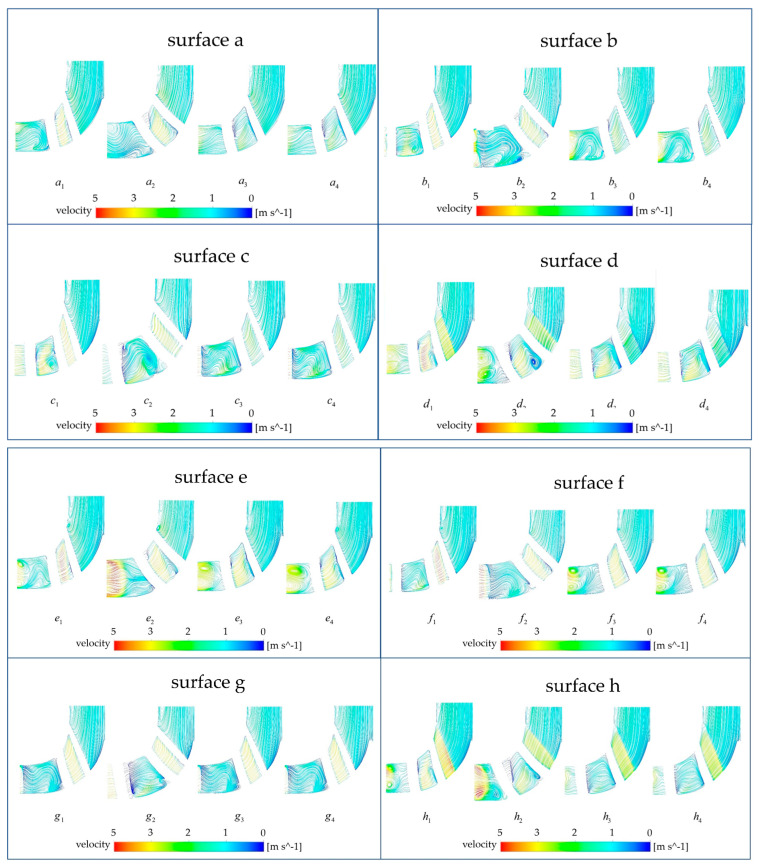
Comparison of monitoring surfaces of the baseline model and the optimized model.

In this study, the three-dimensional streamlines were discretized into two-dimensional streamlines on the monitoring surface to analyze the stream velocity and stream pattern in the impeller calculation domain. Figure 12 illustrates the selection of the monitoring surfaces. A total of eight monitoring surfaces were selected from $a_{i},b_{i},c_{i},\cdot \cdot \cdot \cdot ,h_{i}i\in [1,4]$ (the subscripts *i* from 1 to 4 represent the base model, the A-point model, the B-point model, and the C-point model, respectively) from the axis upward, and the angle between adjacent monitoring surfaces was 45 degrees. Figure 13 shows the stream field comparison of the same monitoring surfaces. In terms of the stream pattern, all models exhibited a stable stream near the impeller inlet with a gentle velocity. This stream pattern changed rapidly to a turbulent pattern when the blood flowed through the impeller. The overall stream chaos was the highest in the A-point model, and an obvious low-speed vortex area could be observed in the monitoring surface of Figure 13 $b_{2}$, $c_{2}$, $d_{2}$. As a result of the large contraction of the rear cover side of the blade in the A-point model, the difference between the front cover side and the rear cover side of the blade in the axial control direction of blood was obvious. Thus, we observed a low-speed stream separation area touching the suction side of the blade in all monitoring surfaces of the A-point model. The overall stream pattern in the B-point and C-point models was stable, and there was no obvious stream separation in the stream channel. During the rotation of monitoring surface a to monitoring surface e, the streamline of the impeller outlet part was gradually distorted, and a local vortex formed at monitoring surfaces e and f (Figure 13 $e_{3}$, $e_{4}$, $f_{3}$, $f_{4}$). As for the rate of stream velocity change, in the stream channel between adjacent blades of the baseline model (Figure 13 $d_{1}$, $e_{1}$, $f_{1}$, $g_{1}$, $h_{1}$), there was a large velocity gradient region, which resulted from the narrowing of the stream channel. This, in turn, was caused by the large blade wrap angle of the baseline model and the crowding-out effect of the blades on the fluid in the stream channel, which increased the stream velocity. The reduction in the wrap angle of the optimized model widened the stream channel between the blades and reduced the bloodstream velocity in the stream channel. As a result, no high-velocity zone was generated in the stream channel of any model after optimization, and only a low-velocity return zone was observed in the axial direction on the $b_{2}$, $d_{2}$ monitoring surface of the A-point model. The velocity variation in the impeller exit region of the A-point model was the most dramatic, and there were obvious high-velocity zones from monitoring surface e to monitoring surface h. The coexistence of the maximum and minimum velocity could even be observed at the monitoring surface of $f_{2}$, $g_{2}$. The overall stream velocity was stable from monitoring surface a to monitoring surface c in the B-point model and C-point model (Figure 13 $a_{3}$, $a_{4}$, $b_{3}$, $b_{4}$, $c_{3}$, $c_{4}$), and only a high-speed vortex area appeared on surface f. Therefore, the blood exhibited a good stream within the B-point model and C-point model. The stream condition in the C-point model was similar to that of the B-point model, but the larger blade area in the C-point model caused a slight increase in the HI value.

**Figure 14:**
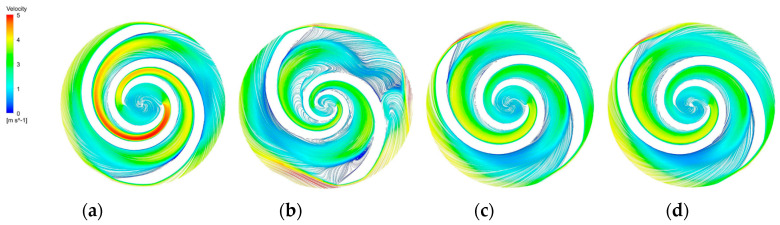
Blade−to−blade cross section at a span = 0.2. (**a**) Blade−to−blade cross section for a span = 0.2 of the base model. (**b**) Blade−to−blade cross section for a span = 0.2 of the A−point model. (**c**) Blade-to-blade cross section for a span = 0.2 of the B−point model. (**d**) Blade−to−blade cross section for a span = 0.2 of the C−point model.

**Figure 15:**
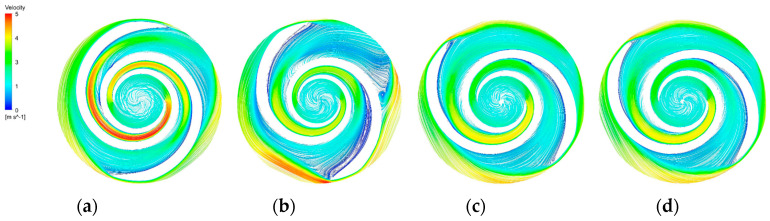
Blade−to−blade cross section at a span = 0.5. (**a**) Blade−to−blade cross section for a span = 0.5 of the base model. (**b**) Blade−to−blade cross section for a span = 0.5 of the A−point model. (**c**) Blade−to−blade cross section for a span = 0.5 of the B−point model. (**d**) Blade−to−blade cross section for a span = 0.5 of the C−point model.

**Figure 16:**
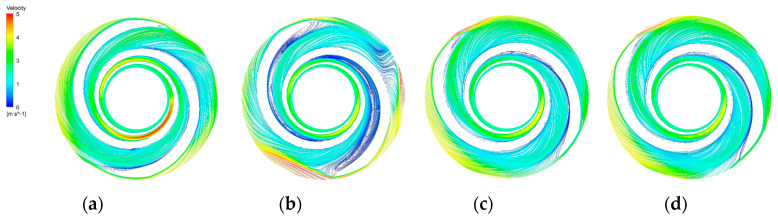
Blade−to−blade cross section at a span = 0.9. (**a**) Blade−to−blade cross section for a span = 0.9 of the base model. (**b**) Blade-to-blade cross section for a span = 0.9 of the A−point model. (**c**) Blade−to−blade cross section for a span = 0.9 of the B−point model. (**d**) Blade−to−blade cross section for a span = 0.9 of the C−point model.

The blade-to-blade sections were intercepted in the radial direction for spans of 0.2, 0.5, and 0.9, respectively. There were more vortices in the A-point model as compared with the other models. In the cross section with a span = 0.2 (Figure 14b), there was a significant backflow in the stream channels of both blade-leading edges for the A-point model. As the impeller diameter increased, the stream velocity in the middle section of the pressure side of the blade decreased, and the streamline spread toward the impeller outlet. The velocity vector on the suction side of the trailing edge of the blade suddenly changed and a stagnation zone was formed. In addition, there were two high stream velocity zones at the gap between the impeller and the volute, which were the same as the monitoring surface e-h, so this was not repeated. In the span = 0.5 cross section (Figure 15b) and the span = 0.9 cross section (Figure 16b), it can be clearly seen that a relatively large low-speed backflow area covered almost all of the pressure surface of the blade, indicating that the most serious area of backflow occurred on the front cover side of the blade working surface. From the three cross sections of the B-point model (Figure 14c, Figure 15c and Figure 16c), it can be seen that the streamlines within the impeller of the B-point model were uniformly distributed and close to the blade profile. Moreover, they fit closely to the blade and there was no stream separation region in the blade suction surface. In addition, the stream velocity in the stream channel was controlled in the range of 1 m/s~4 m/s, and the stable stream pattern minimized the destruction of red blood cells in the stream channel. Three radial sections of the C-point model exhibited similar stream conditions as those of the B-point model, but in the span = 0.5 and 0.9 sections (Figure 15d and Figure 16d), the left blade suction surface had fewer low-velocity areas and reflux areas (Figure 15c and Figure 16c), which meant the C-point model had the lowest risk of thrombosis.

### 3.4. SSS and HI Index Analysis

**Figure 17:**
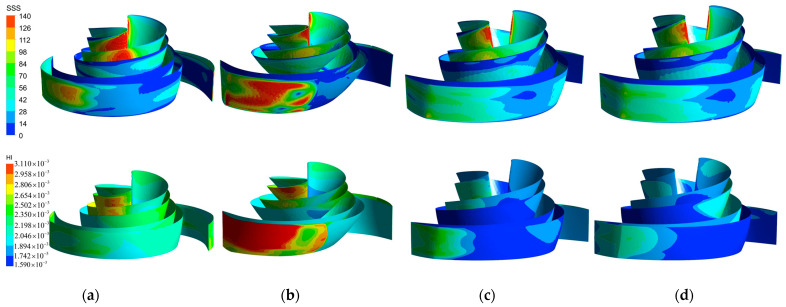
SSS distribution and HI distribution. (**a**) SSS distribution and HI distribution of the baseline model. (**b**) SSS distribution and HI distribution of the A−point model. (**c**) SSS distribution and HI distribution of the B−point model. (**d**) SSS distribution and HI distribution of the C−point model.

In the HI index formula, the SSS value dominates, so the distribution of SSS correlates with the HI value distribution of the blood pump. Figure 17 compares the SSS distribution clouds on the blade surfaces of the four models and the HI index distribution clouds. In all models, the SSS on the pressure surface of the blade was generally higher than that on the suction surface. The majority of the HI values of the baseline model blade (Figure 17a) were between 1.894 × 10^−3^ and 2.502 × 10^−3^, and the high SSS region (100 Pa–140 Pa) was mainly concentrated in the flow path near the leading edge of the blade. Thus, there was a local high HI region at the same location on the HI distribution map. The high SSS region and the high HI region of the A-point model were the most widely distributed. The larger blade area and the chaotic flow pattern on the pressure side of the blade (Figure 15b) led to a sudden increase in SSS at the leading and trailing edges of the front cover side of the blade (Figure 17b), so a large high SSS region (>100 Pa) and a large HI region covered the entire trailing edge of the blade. The B-point model (Figure 17c) exhibited the smallest high SSS region, except for the high shear region at the leading edge of the blade, with SSS in the rest of the parts remaining below 100 Pa. From the HI distribution, it can be seen that the middle section of the blade caused the least damage to the blood. This was essentially below 1.742 × 10^−3^. In the leading and trailing edges of the blade, the HI value rose to 2.654 × 10^−3^. The C-point model maintained a similar HI distribution and SSS distribution as the B-point model, but the degree of blood damage at the impeller inlet was slightly higher than that of the B-point model due to the difference in the shape of the leading edge of the blade.

**Figure 18:**
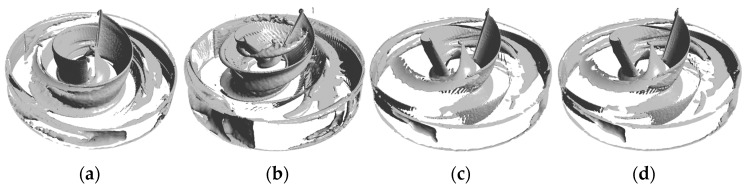
Vortex distribution. (**a**) Vortex distribution of the baseline model. (**b**) Vortex distribution of the A-point model. (**c**) Vortex distribution of the B-point model. (**d**) Vortex distribution of the C-point model.

Finally, in this study, the vortex situation in all model fluid domains was analyzed using a vortex identification method based on the Q-criterion. This is, in turn, based on the second matrix invariant of the velocity gradient tensor [56] and Equation (15):

(15) $$Q=\frac{1}{2}({\left\|B\right\|}_{F}^{2}-{\left\|A\right\|}_{F}^{2})$$

where ${\left\|\right\|}_{F}$ denotes the Frobenius parametrization of the matrix, and A and B represent the deformation and rotation of a point in the flow field, respectively. In this study, *Q* = 0.01 was used to identify the vortex in the impeller domain, as shown in Figure 18. The figure shows that most of the vortices for all models were concentrated on the blade surface and the impeller exit, and the vortex distribution in the blade mid-working surface and trailing edge of the A-point model (Figure 18b) was denser. Vortices still existed on the blade leading-edge surface for the B-point model and the C-point model, but the density of vortices at the exit and in the flow channel was significantly lower.

## 4. Discussion

Related studies have confirmed that hemolysis is an important cause of blood damage in blood pumps [57,58,59]. In the published literature, most scholars reduce the HI value in blood pumps and thrombus by manually adjusting the impeller and volute parameters [60,61,62]. However, trial-and-error analysis and numerical simulations can be time-consuming and ignore the co-influence of the impeller parameters on the hydraulic performance and HI performance, which may result in bypassing the optimal combined solution. The multi-objective optimization algorithm can obtain a set of optimal solutions in a short time and select the final optimization result independently according to the requirements, which greatly improves optimization efficiency and speed. In this study, we developed an optimization process for a screw centrifugal blood pump using a multi-algorithm machine-learning approach. The whole process is divided into three parts: random forest algorithm prediction, MOGWO optimization, and internal flow field analysis.

During the random forest algorithm prediction stage, 240 sets of impeller parameters were randomly generated using the Latin hypercube sampling method. Then, these were modeled and numerically simulated to obtain a dataset consisting of impeller parameters, pressure generation, and HI values. Thereafter, two independent random forest prediction models were established with impeller parameters as input values, and pressure generation and HI values as output values. These were used as the objective functions in the multi-objective optimization algorithm. During the multi-objective optimization stage, the multi-objective gray wolf optimization algorithm was selected as the optimizer for this study. The model that exhibited the highest-pressure generation, the model that exhibited the lowest HI value, and the middle point model were selected as the optimized models in the Pareto front with iterations up to 400 generations.

Finally, the hydraulic performance and HI performance of all blood pumps were compared using pressure cloud, velocity flow line, SSS distribution, HI distribution, and vortex distribution plots. In terms of hydraulic performance, all optimized models exhibited different degrees of pressure generation increase. The A-point model had the largest pressure generation increase because the maximum vane outlet angle and vane outlet width provided the largest effective work area. The B-point model and C-point model demonstrated a lesser pressure generation increase than the A-point model, but the C-point model exhibited a 5% higher pump pressure generation than the B-point model. Thus, the C-point model had a higher hydraulic efficiency than the B-point model. The hydraulic efficiency of the C-point model was higher than that of the B-point model. With regards to HI performance, a power law equation based on SSS and exposure time was used to assess the blood compatibility of the blood pump. In the baseline model, the overall flow state of the blood was smooth, but a high shear zone caused by a high-flow velocity existed in the flow channel near the leading edge of the impeller, resulting in a high HI value at this location. The inlet side of the blade in the A-point model extended significantly forward along the rear cover plate profile, the hub side of the blade shrank toward the center, and the irregular impeller shape led to a large flow separation and backflow near the working surface of the blade, which elevated the thrombus formation risk. In the radial flow diagram, it can be observed that the flow separation area with the large velocity gradient was touching the middle and trailing edge of the impeller, so the blade of the A-point model had the largest high SSS area and high HI area, and the blood damage degree of the A-point model was the most serious. The B-point model blade on the inlet side, along the front cover plate profile forward extension, caused the blade to be biased to the axial placement. Each height of the blade was similar in terms of the direction of action to the blood, so the flow pattern was stable and close to the blade surface. In addition, the spacious flow channel caused a small change in the flow velocity and no flow separation in the flow field. The majority of the blade SSS was maintained below 100 Pa. In the HI distribution, the HI values of all parts of the blade were below 2.654 × 10^−3^. The SSS and HI distributions of the C-point model were similar to those of the B-point model, but the HI values were 2% higher than those of the B-point model. This may indicate that the increase in the HI value for the C-point model originated from the increase in the impeller surface area rather than from the difference in the impeller shape, and if the pressure generation of the C-point model was reduced to the same value as that of the B-point model by reducing the speed, its HI value would decrease accordingly. In summary, the C-point model exhibited the largest increase in pressure generation while reducing the HI value under the same operating conditions. Thus, it is the best solution among all the optimized models.

There are some limitations to this study. First, because the impeller diameter and the base circle diameter of the pressurized water chamber were fixed during the design process, the width of the gap between the blade and the volute sidewall was not included as a design variable. In fact, the width of the gap also had an impact on the HI level [22]. In addition, the friction between the rotor and the stator during rotation in the blood pump generated thermal effects, which may have also had an impact on the red blood cells. In this study, the thermal effect was not considered during the simulation.

## 5. Conclusions

This study provides a complete multi-objective optimization process for a spiral centrifugal blood pump. The process contains model parameterization, Latin hypercube sampling, random forest prediction, a numerical simulation, and a multi-objective gray wolf optimization algorithm. In the first step, six impeller parameters are selected to control the shape of the impeller. Then, 240 sets of impeller parameters are randomly generated using the Latin hypercube sampling method, and these are numerically simulated to obtain a database for training the random forest prediction model. In the second step, the Pareto front is calculated using the multi-objective gray wolf optimization algorithm, and the highest point of the lift model (the A-point model), the lowest point of the HI model (the B-point model), and the middle point model (the C-point model) are selected as the optimized models. In the last step, the pressure cloud, the velocity flow line, the SSS distribution, the HI distribution, and the vortex distribution of all the optimized models are compared with the baseline model. The comparison results show that the pressure generation in the A-point model has increased by 41% and the HI value has increased by 105%. The pressure generation in the B-point model has increased by 19% and the HI value has decreased by 50%. The pressure generation in the C-point model has increased by 24% and the HI value has decreased by 48%, and the increase in the HI value is almost negligible as compared with the increase in pressure generation. Thus, the C-point model is the best-optimized model. The coupled optimization algorithm proposed in this paper exhibited a high prediction accuracy and fast optimization speed and can be used as a highly efficient optimization method for screw centrifugal blood pumps. In future research, the method proposed in this paper can be used to optimize other types of left ventricular assist devices such as axial flow pumps.

## Figures and Tables

**Table 1:** Impeller parameter values of the base model and the range of impeller parameter variations.

Design Variables	Unit	Baseline Model	$x_{L}$	$x_{U}$
$b_{2}$	mm	4.9	4.5	6
$\beta_{2}$	degree	23.3	15	45
ϕ	degree	460	400	500
$u_{1}$	mm	3.3	2	5
$u_{2}$	mm	11.8	10	12.5
$A_{2}$	-	8.1	1	10
dS	mm	10	-	-
$d_{2}$	mm	30	-	-

**Table 2:** Mesh-independent verification.

Programs	Number of Meshes	Pressure Generation
1	845,279	81.5
2	1,747,747	96.4
3	3,825,107	102.2
4	5,137,205	103.1

**Table 3:** Prediction model accuracy evaluation table.

	HI Prediction Set	Pressure Prediction Set
MSE	0.34	0.003
MAPE	0.13	0.05

**Table 4:** Comparison of impeller parameters of the A-point model, the B-point model, the C-point model, and the baseline model.

Design Variable	Unit	Baseline Model	A-Point Model	B-Point Model	C-Point Model
$b_{2}$	mm	4.9	6	5.2	5.5
$\beta_{2}$	degree	23.3	23.3	22	23
ϕ	degree	460	433	400	400
$u_{1}$	mm	3.3	3.1	2.7	2.6
$u_{2}$	mm	11.8	10.1	10.9	11.1
$A_{r}$	-	8.1	7.9	8.3	7.7

## Data Availability

The data presented in this article are available upon request from the corresponding author.

## References

[1] Townsend N., Kazakiewicz D., Lucy Wright F., Timmis A., Huculeci R., Torbica A., Gale C.P., Achenbach S., Weidinger F., Vardas P. (2020). Epidemiology of cardiovascular disease in Europe. Nat. Rev. Cardiol..

[2] Savarese G., Lund L.H. (2017). Global public health burden of heart failure. Card. Fail. Rev..

[3] Savarese G., Becher P.M., Lund L.H., Seferovic P., Rosano G.M., Coats A.J. (2022). Global burden of heart failure: A comprehensive and updated review of epidemiology. Cardiovasc. Res..

[4] Colvin M., Smith J.M., Hadley N., Skeans M.A., Uccellini K., Goff R., Kasiske B.L. (2020). OPTN/SRTR 2018 annual data report: Heart. Am. J. Transplant..

[5] Ahmad T., Patel C.B., Milano C.A., Rogers J.G. (2012). When the heart runs out of heartbeats: Treatment options for refractory end-stage heart failure. Circulation.

[6] Miller L.W., Pagani F.D., Russell S.D., John R., Boyle A.J., Aaronson K.D., Frazier O. (2007). Use of a continuous-flow device in patients awaiting heart transplantation. N. Engl. J. Med..

[7] Jing T., Xu H., Wang H., Wang F., Qian K. (2012). Experiment of transcutaneous energy transmission system for heart pump. J. Jiangsu Univ..

[8] Jing T., Pan A., Gu F., Wang X. (2022). Numerical simulation and hemolysis analysis of aortic perforating type axial bleeding pump with folded-edge structure impeller. J. Drain. Irrig. Mach. Eng..

[9] Jing T., Gu L., Wang F., He Z. (2020). Analysis of speed and internal flow field of axial flow blood pump in optimal left heart assistance. J. Drain. Irrig. Mach. Eng..

[10] O’Brien C., Monteagudo J., Schad C., Cheung E., Middlesworth W. (2017). Centrifugal pumps and hemolysis in pediatric extracorporeal membrane oxygenation (ECMO) patients: An analysis of Extracorporeal Life Support Organization (ELSO) registry data. J. Pediatr. Surg..

[11] O’Halloran C.P., Thiagarajan R.R., Yarlagadda V.V., Barbaro R.P., Nasr V.G., Rycus P., Alexander P.M. (2019). Outcomes of infants supported with extracorporeal membrane oxygenation using centrifugal versus roller pumps: An analysis from the ELSO registry. Pediatr. Crit. Care Med. A J. Soc. Crit. Care Med. World Fed. Pediatr. Intensive Crit. Care Soc..

[12] Johnson K.N., Carr B., Mychaliska G.B., Hirschl R.B., Gadepalli S.K. (2022). Switching to centrifugal pumps may decrease hemolysis rates among pediatric ECMO patients. Perfusion.

[13] Fox C.S., Palazzolo T., Hirschhorn M., Stevens R.M., Rossano J., Day S.W., Throckmorton A.L. (2022). Development of the centrifugal blood pump for a hybrid continuous flow pediatric total artificial heart: Model, make, measure. Front. Cardiovasc. Med..

[14] Selmi M., Chiu W.C., Chivukula V.K., Melisurgo G., Beckman J.A., Mahr C., Consolo F. (2019). Blood damage in Left Ventricular Assist Devices: Pump thrombosis or system thrombosis?. Int. J. Artif. Organs.

[15] Reul H.M., Akdis M. (2000). Blood pumps for circulatory support. Perfusion.

[16] Feldmann C., Zayat R., Goetzenich A., Aljalloud A., Woelke E., Maas J., Moza A. (2017). Perioperative onset of acquired von Willebrand syndrome: Comparison between HVAD, HeartMate II and on-pump coronary bypass surgery. PLoS ONE.

[17] Ghadimi B., Nejat A., Nourbakhsh S.A., Naderi N. (2019). Shape optimization of a centrifugal blood pump by coupling CFD with metamodel-assisted genetic algorithm. J. Artif. Organs.

[18] Olia S.E., Maul T.M., Antaki J.F., Kameneva M.V. (2016). Mechanical blood trauma in assisted circulation: Sublethal RBC damage preceding hemolysis. Int. J. Artif. Organs.

[19] Hosseini S.E., Keshmiri A. (2022). Experimental and numerical investigation of different geometrical parameters in a centrifugal blood pump. Res. Biomed. Eng..

[20] Wiegmann L., Boës S., de Zélicourt D., Thamsen B., Schmid Daners M., Meboldt M., Kurtcuoglu V. (2018). Blood pump design variations and their influence on hydraulic performance and indicators of hemocompatibility. Ann. Biomed. Eng..

[21] Li Y., Wang H., Xi Y., Sun A., Deng X., Chen Z., Fan Y. (2023). Impact of volute design features on hemodynamic performance and hemocompatibility of centrifugal blood pumps used in ECMO. Artif. Organs.

[22] Li Y., Bai C., Qiao H., Yang Y. (2022). Flow characteristics of viscous oil in rotor cavity of cam pump. J. Jiangsu Univ..

[23] Onder A., Incebay O., Sen M.A., Yapici R., Kalyoncu M. (2021). Heuristic optimization of impeller sidewall gaps-based on the bees algorithm for a centrifugal blood pump by CFD. Int. J. Artif. Organs.

[24] Antaki J.F., Ghattas O., Burgreen G.W., He B. (1995). Computational flow optimization of rotary blood pump components. Artif. Organs.

[25] Deb K., Burke E.K., Kendall G. (2005). Multi-objective optimization. Search Methodologies.

[26] Zhu L., Zhang X., Yao Z. (2010). Shape optimization of the diffuser blade of an axial blood pump by computational fluid dynamics. Artif. Organs.

[27] Li R., Li B., Han W. (2005). Analysis on working characteristics of screw centrifugal pump. Nongye Jixie Xuebao.

[28] Nazarenko H. (2021). Analytical and Experimental Assessment Of Screw Centrifugal Pump At Improving Its Design. Natsional’nyi Hirnychyi Universytet. Nauk. Visnyk.

[29] Cheng X., Li R. (2012). Parameter equation study for screw centrifugal pump. Procedia Eng..

[30] Zhang X.Z., Wang Y.W., Hu J.S. (2019). Parameter optimization of centrifugal pump impeller. IOP Conference Series: Materials Science and Engineering.

[31] Feng K., Song P., Chen Y., Liu H., Li X. (2019). Drawing and inspection of the axial projection view of the centrifugal pump impeller. J. Phys. Conf. Ser..

[32] Mozafari S., Rezaienia M.A., Paul G.M., Rothman M.T., Wen P., Korakianitis T. (2017). The effect of geometry on the efficiency and hemolysis of centrifugal implantable blood pumps. Asaio J..

[33] Ozturk C., Aka I.B., Lazoglu I. (2018). Effect of blade curvature on the hemolytic and hydraulic characteristics of a centrifugal blood pump. Int. J. Artif. Organs.

[34] Luo H., Tao R., Yang J., Wang Z. (2020). Influence of blade leading-edge shape on rotating-stalled flow characteristics in a centrifugal pump impeller. Appl. Sci..

[35] Garud S.S., Karimi I.A., Kraft M. (2017). Design of computer experiments: A review. Comput. Chem. Eng..

[36] McKay M.D., Beckman R.J., Conover W.J. (2000). A comparison of three methods for selecting values of input variables in the analysis of output from a computer code. Technometrics.

[37] Stein M. (1987). Large sample properties of simulations using Latin hypercube sampling. Technometrics.

[38] Mazumdar J. (2015). Biofluid Mechanics.

[39] Chen Z., Jena S.K., Giridharan G.A., Sobieski M.A., Koenig S.C., Slaughter M.S., Wu Z.J. (2019). Shear stress and blood trauma under constant and pulse-modulated speed CF-VAD operations: CFD analysis of the HVAD. Med. Biol. Eng. Comput..

[40] Denisov M.V., Telyshev D.V., Selishchev S.V., Romanova A.N. (2019). Investigation of hemocompatibility of rotary blood pumps: The case of the sputnik ventricular assist device. Biomed. Eng..

[41] Ye W., Geng C., Luo X. (2022). Unstable flow characteristics in vaneless region with emphasis on the rotor-stator interaction for a pump turbine at pump mode using large runner blade lean. Renew. Energy.

[42] Rodi W. (2017). Turbulence Models and Their Application in Hydraulics: A State-of-the-Art Review.

[43] Han Y., Zhou L., Bai L., Shi W., Agarwal R. (2021). Comparison and validation of various turbulence models for U-bend flow with a magnetic resonance velocimetry experiment. Phys. Fluids.

[44] Zhang J., Gellman B., Koert A., Dasse K.A., Gilbert R.J., Griffith B.P., Wu Z.J. (2006). Computational and experimental evaluation of the fluid dynamics and hemocompatibility of the CentriMag blood pump. Artif. Organs.

[45] Yu H., Janiga G., Thévenin D. (2016). Computational fluid dynamics-based design optimization method for archimedes screw blood pumps. Artif. Organs.

[46] Karimi M.S., Razzaghi P., Raisee M., Hendrick P., Nourbakhsh A. (2021). Stochastic simulation of the FDA centrifugal blood pump benchmark. Biomech. Model. Mechanobiol..

[47] Taskin M.E., Fraser K.H., Zhang T., Wu C., Griffith B.P., Wu Z.J. (2012). Evaluation of Eulerian and Lagrangian models for hemolysis estimation. ASAIO J..

[48] Giersiepen M., Wurzinger L.J., Opitz R., Reul H. (1990). Estimation of shear stress-related blood damage in heart valve prostheses-in vitro comparison of 25 aortic valves. Int. J. Artif. Organs.

[49] Bludszuweit C. (1995). Model for a general mechanical blood damage prediction. Artif. Organs.

[50] Breiman L. (2001). Random forests. Mach. Learn..

[51] Segal M.R. (2004). Machine Learning Benchmarks and Random Forest Regression Center for Bioinformatics and Molecular Biostatistics.

[52] De Myttenaere A., Golden B., Le Grand B., Rossi F. (2016). Mean absolute percentage error for regression models. Neurocomputing.

[53] Allen D.M. (1971). Mean square error of prediction as a criterion for selecting variables. Technometrics.

[54] Mirjalili S., Mirjalili S.M., Lewis A. (2014). Gray wolf optimizer. Adv. Eng. Softw..

[55] Mirjalili S., Saremi S., Mirjalili S.M., Coelho L.D.S. (2016). Multi-objective grey wolf optimizer: A novel algorithm for multi-criterion optimization. Expert Syst. Appl..

[56] Zhang Y.N., Qiu X., Chen F.P., Liu K.H., Dong X.R., Liu C. (2018). A selected review of vortex identification methods with applications. J. Hydrodyn..

[57] Seki H., Fujiwara T., Hijikata W., Murashige T., Tahara T., Yokota S., Arai H. (2021). Evaluation of real-time thrombus detection method in a magnetically levitated centrifugal blood pump using a porcine left ventricular assist circulation model. Artif. Organs.

[58] Rowlands G.W., Antaki J.F. (2020). High-speed visualization of ingested, ejected, adherent, and disintegrated thrombus in contemporary ventricular assist devices. Artif. Organs.

[59] Zhou L., Hang J., Bai L., Krzemianowski Z., El-Emam M.A., Yasser E., Agarwal R. (2022). Application of entropy production theory for energy losses and other investigation in pumps and turbines: A review. Appl. Energy.

[60] Aka I.B., Ozturk C., Lazoglu I. (2021). Numerical investigation of volute tongue design on hemodynamic characteristics and hemolysis of the centrifugal blood pump. SN Appl. Sci..

[61] Huang B., Guo M., Lu B., Wu Q., Zuo Z., Liu S. (2021). Geometric Optimization of an Extracorporeal Centrifugal Blood Pump with an Unshrouded Impeller Concerning Both Hydraulic Performance and Shear Stress. Processes.

[62] Wang L., Yun Z., Tang X., Xiang C. (2022). Influence of circumferential annular grooving design of impeller on suspended fluid force of axial flow blood pump. Int. J. Artif. Organs.

